# A Quantitative Analysis of the Impact on Chromatin Accessibility by Histone Modifications and Binding of Transcription Factors in DNase I Hypersensitive Sites

**DOI:** 10.1155/2013/914971

**Published:** 2013-10-22

**Authors:** Peng Cui, Jing Li, Bo Sun, Menghuan Zhang, Baofeng Lian, Yixue Li, Lu Xie

**Affiliations:** ^1^School of Life Science and Biotechnology, Shanghai Jiao Tong University, 800 Dong Chuan Road, Shanghai 200240, China; ^2^Shanghai Center for Bioinformation Technology, 1278 Ke Yuan Road, Shanghai 201203, China

## Abstract

It is known that chromatin features such as histone modifications and the binding of transcription factors exert a significant impact on the “openness” of chromatin. In this study, we present a quantitative analysis of the genome-wide relationship between chromatin features and chromatin accessibility in DNase I hypersensitive sites. We found that these features show distinct preference to localize in open chromatin. In order to elucidate the exact impact, we derived quantitative models to directly predict the “openness” of chromatin using histone modification features and transcription factor binding features, respectively. We show that these two types of features are highly predictive for chromatin accessibility in a statistical viewpoint. Moreover, our results indicate that these features are highly redundant and only a small number of features are needed to achieve a very high predictive power. Our study provides new insights into the true biological phenomena and the combinatorial effects of chromatin features to differential DNase I hypersensitivity.

## 1. Introduction

In eukaryotes, DNA is organized into chains of nucleosomes, each of which consists of about 146 bp of DNA wrapped around an octamer of four types of histones [1]. The packaging of chromatin into nucleosomes provides a repressive environment for many DNA-binding proteins and plays an important role in the regulation of transcription [2]. However, some domains in chromatin are depleted of nucleosomes and exhibit highly accessible structure. These nucleosome-free regions are supersensitive to the cleavage of DNase I [3] and are known as DNase I hypersensitive sites (DHSs). They are predominantly found in many active genes and cis-regulatory elements [4]. The dynamic alterations of “openness” in chromatin play important roles in many biological processes, including transcription [5], replication [2], and differentiation [6].

Traditionally, the experimental technique of choice to discover the DNase I hypersensitive sites is Southern blotting [7]. However, this low-throughput method is not able to study large chromosomal regions at a time and cannot represent the “openness” of chromatin in a quantitative manner. The significance of differential accessibility in DNase I hypersensitive sites is unknown, but it may reflect some important biological phenomena like histone modifications and protein occupation [8]. Even until now genome-wide quantitative analyses of the relationship between chromatin accessibility and chromatin features in DNase I hypersensitive sites are rare. By taking advantage of the abundant datasets of the ENCODE project [9], we analyzed genome-wide localization data of DNase I hypersensitive sites and 33 chromatin features in human embryonic stem cell (H1hesc) cell line. All datasets were generated by recently developed genome-wide high throughput experimental techniques, such as Chip-seq [10, 11] and DNase-seq [12].

It is generally accepted that histone modifications and the binding of transcription factors are two main effectors for the “openness” of chromatin. Previous studies have shown that histone modifications and transcription factors tend to occur near or just in the DNase I hypersensitive sites [8, 13]. Recently, two studies, one in K562 cell line and the other in Drosophila embryonic cells, have demonstrated that transcription factor binding sites and the chromatin accessibility are highly correlated with each other [6, 13]. Although these studies provided important information, so far, quantitative analysis of the combinatorial effects of different chromatin features and the biological significance of differential hypersensitivity is still unclear. In this work, we built support vector regression (SVR) models to directly predict the “openness” of chromatin in DNase I hypersensitive sites using combined chromatin features. Our results indicate that both histone modification features and transcription factor binding features are predictive for chromatin accessibility with high accuracy and these chromatin features are highly redundant.

## 2. Materials and Methods 

### 2.1. Datasets

All datasets are from ENCODE project, which aims to build a comprehensive list of functional elements in the human genome [9]. The 10 histone modifications (HMs) and binding sites of 23 transcription factors (TFs) were quantified using Chip-seq and downloaded from the tracks of UCSC Genome Browser at ENCODE/Broad Institute and ENCODE/Stanford/Yale/USC/Harvard. The chromatin accessibility dataset was measured using DNase-seq and downloaded from ENCODE/OpenChrom (Duke/UNC/UTA). Each dataset includes the genome-wide sequencing signals and regions of statistically enriched signal (peaks). Peaks can be viewed as locations of chromatin features and DNase I hypersensitive sites, respectively, and the values of DNase-seq signals represent chromatin accessibility. We must note that DNase I hypersensitivity could not be simply viewed as binary property (peaks versus nonpeaks) but rather continuous values (sequencing signals) representing differential chromatin accessibility. These datasets come from the common H1hesc cell line.

### 2.2. Mapping HM and TF Binding Peaks to the DNase I Hypersensitive Sites

We obtained genomic locations of 33 chromatin feature profiles, all together including 582489 histone modification peaks (10 HMs) and 443217 transcription factor binding peaks (23 TFs). For each profile, we mapped the peaks of feature onto the genome and examined whether it localized in open chromatin or not. The presence or absence of chromatin feature within accessible chromatin was decided by overlap or nonoverlap with DNase-seq peaks. If there was any amount of overlap within accessible chromatin (DNase-seq peaks), we counted as a presence [13]. Then, we calculated the percentage of the peaks occurring in the DNase I hypersensitive sites for each feature.

### 2.3. Supervised Learning Methods for Chromatin Accessibility Prediction

To investigate the quantitative relationship between chromatin accessibility and these chromatin features in DNase I hypersensitive sites, we constructed support vector regression (SVR) models for HM and TF binding features, respectively. Concretely, in every DNase I hypersensitive site, we calculated the maximum signal of DNase-Seq and the corresponding maximum signal of Chip-Seq for each chromatin feature. For the sake of figuring out whether the maximum signal exhibits largest prediction power or not, as a comparison, we also calculated the average signal of Chip-seq and DNase-seq for each hypersensitive region. Then, SVR model was built to predict the chromatin accessibility using signals of these chromatin features. SVR is a machine learning algorithm based on statistical theory for regression problems [14, 15]. We implemented this algorithm using the “e1071” *R* package [16].

In order to reduce the computation cost, we randomly selected 5000 DHSs for our samples. The sample size analysis indicated that the prediction power increased only moderately after the size reached 2000. So, the sample size of 5000 is big enough to represent the entire dataset (Supporting Information S1 which is available online at http://dx.doi.org/10.1155/2013/914971). We used the 10-fold cross-validation method to evaluate the prediction power. Specifically, we randomly split our sample dataset into 10 equal size subsets. Among them, 9 subsets were used as training data and the remaining subset was treated as the validation data for testing the model. This process was repeated 10 times and each subset could only be used once as the validation data. After that, we combined the results and plotted the regression relationship between predicted signals and the actual DNase-seq signals. Then, the coefficient of determination (*R* ^2^) [17] was computed indicating how well these data points fit the line. *R* ^2^ is also a frequently used measure of the proportion of total variation of outcomes explained by the model. We chose the square root of the coefficient of determination (*R*) as our prediction power.

### 2.4. Analysis of the Importance for Each Chromatin Feature and the Combinatorial Effects of Different Features

To estimate which feature exhibits the maximal prediction power, we predicted the chromatin accessibility using only one feature. And to investigate whether HM features and TF binding features are redundant, we next predicted the “openness” of chromatin using all features. We also explored the combinatorial effects of these features. All possible one-feature (C_33_^1^), two-feature (C_33_^2^), and three-feature (C_33_^3^) models were evaluated by their performance.

### 2.5. Model Comparison Analysis

Instead of SVR algorithm, we also explored the quantitative relationship between chromatin features and chromatin accessibility with linear regression model. Similarly, HM features, TF binding features, and HM+TF feature combinations were applied to linear regression model, respectively. The coefficient of determination of the predicted signals and the actual DNase-seq signals were calculated and compared with the SVR models. In order to identify whether the maximum signals or the average signals exhibit largest prediction power, we also applied these models with the average signals of chromatin features to predict the average signals of DNase-seq.

## 3. Results 

### 3.1. The Localization Preference of Chromatin Features

**Figure 1:**
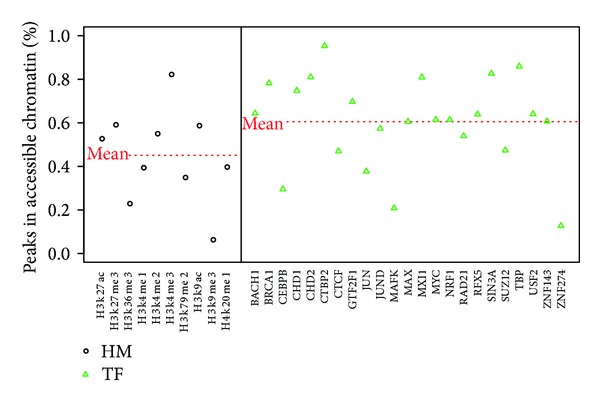
The percentages of histone modification (HM) features and transcription factor (TF) binding features within accessible chromatin regions. The black circle and green triangle represent HM features and TF binding features, respectively. The two red lines represent the mean percentages for HMs and TFs, respectively.

We analyzed genome-wide localizations of 33 Chip-seq profiles in the human embryonic stem cell line (H1hesc) from ENCODE project [9], including 10 histone modifications, and the binding sites of 23 transcription factors. For each profile, we mapped the peaks of Chip-seq dataset to the DNase I hypersensitive sites (see Section 2).  Figure 1 shows the percentage of the peaks within the accessible chromatin for each feature. We observed that different chromatin feature exhibits different preference to chromatin accessibility. For histone modifications, H3k4me3 exerts the largest preference of accessible chromatin. 82.2% H3k4me3 peaks located in DHS. On the contrary, most H3k9me3 occurred out of DHS (93.7%), which indicated that H3k9me3 was associated with heterochromatin [18]. Compared to histone modifications, a majority of transcription factors tend to bind onto accessible chromatin, which suggests that the process of transcription requires an open chromatin structure [19]. The mean percentage of transcription factors locating in DHS is 60.5%, higher than that of histone modifications (45.1%).

### 3.2. Predicting Chromatin Accessibility Using Histone Modification Features

**Figure 2:**
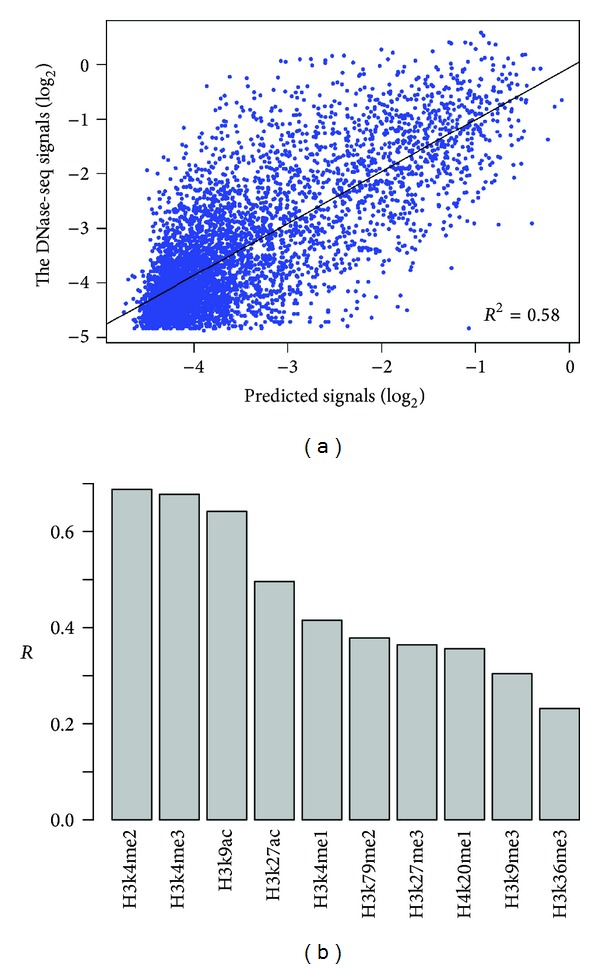
Prediction accuracy of chromatin accessibility using HM features. (a) Scatter plot of predicted versus experimentally measured DNase-seq signals using all HM features. The black line represents the linear fit between predicted and measured signals (*R* ^2^, coefficient of determination). (b) Prediction powers (*R*, the square root of coefficient of determination) of the SVR models using only one particular HM feature.

In order to examine the quantitative relationship between chromatin accessibility and HM features in a combinatorial manner, we constructed SVR model to predict the “openness” of chromatin in DNase I hypersensitive sites using all histone modification features. We can see from Figure 2(a) that there is a linear relationship between predicted signals and the actual DNase-Seq signals. The coefficient of determination (*R* ^2^) is 0.58 indicating that histone modification features explain about 58% variance of chromatin accessibility. 

We next examined the prediction power for every histone modification feature.  Figure 2(b) shows that H3k4me2, H3k4me3, and H3k9ac exhibit the most important effects to chromatin accessibility (*R* = 0.67, 0.66,0.63, resp.). These histone modifications are generally enriched in the promoters of expressed genes [20] and the open chromatin structure plays an important role in regulating the complex transcription process. On the other hand, H3k9me3 and H3k36me3 exhibit the least prediction powers (*R* = 0.30,0.23, resp.), which suggests that these modifications are associated with heterochromatin [21, 22]. Interestingly, H3k27ac and H4k20me1, which are the most predictive histone modifications for gene expression levels [23], are not the most important features associated with chromatin accessibility.

### 3.3. Predicting Chromatin Accessibility Using Transcription Factor Binding Features

**Figure 3:**
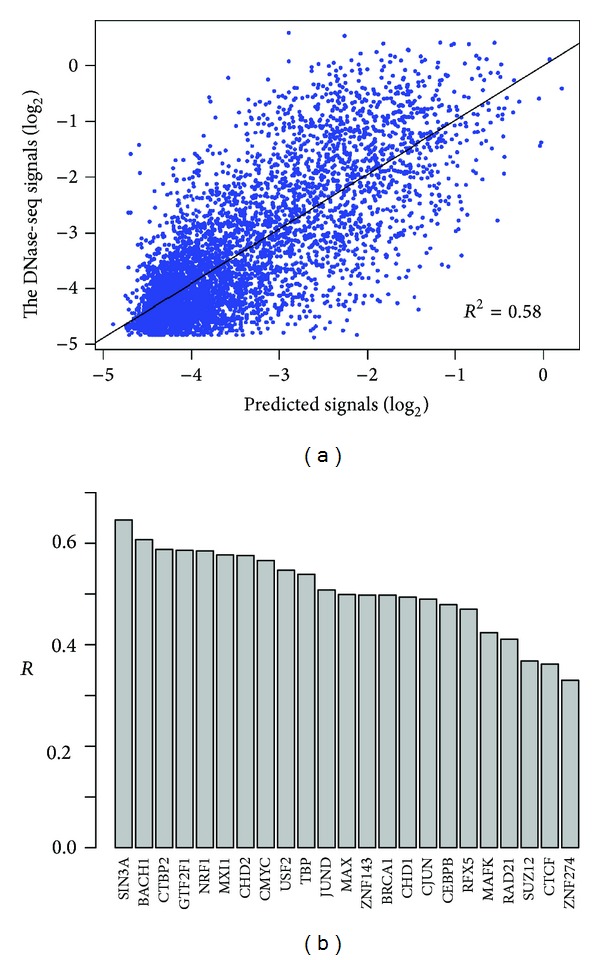
Prediction accuracy of chromatin accessibility using TF binding features. (a) Scatter plot of predicted versus experimentally measured DNase-seq signals using all TF binding features. The black line represents the linear fit between predicted and measured signals (*R* ^2^, coefficient of determination). (b) Prediction powers (*R*, the square root of coefficient of determination) of the SVR models using only one particular TF binding feature.

Previous studies have shown that transcription factors tend to bind onto open chromatin and they are highly correlated with each other [6, 13]. To investigate the quantitative relationship of the binding of transcription factors and the chromatin accessibility in a combinatorial manner, we next applied our SVR model to all TF binding features. As shown in Figure 3(a), the TF model achieves a coefficient of determination (*R* ^2^) of 0.58 which is equal to that achieved by HM model. These TF binding features can also explain about 58% variance of chromatin accessibility.

For the prediction power of particular TF binding feature, there is a difference with that of histone modifications; that is, most transcription factors exhibit important effects to chromatin accessibility (Figure 3(b)). This is consistent with their functions because transcription factors directly control the complex transcription process [24] which requires an open chromatin environment. However, a small group of features exhibit lower prediction powers, such as SUZ12, CTCF, and ZNF274 (*R* = 0.37,0.36,0.33, resp.). ZNF274 and SUZ12 are known to be transcriptional repressors [25, 26]. CTCF has many roles, such as transcriptional repression, insulator function, and imprinting genetic information [27]. These factors are not so important to contribute to the “openness” of chromatin.

### 3.4. Chromatin Features Are Highly Redundant to Chromatin Accessibility

**Figure 4:**
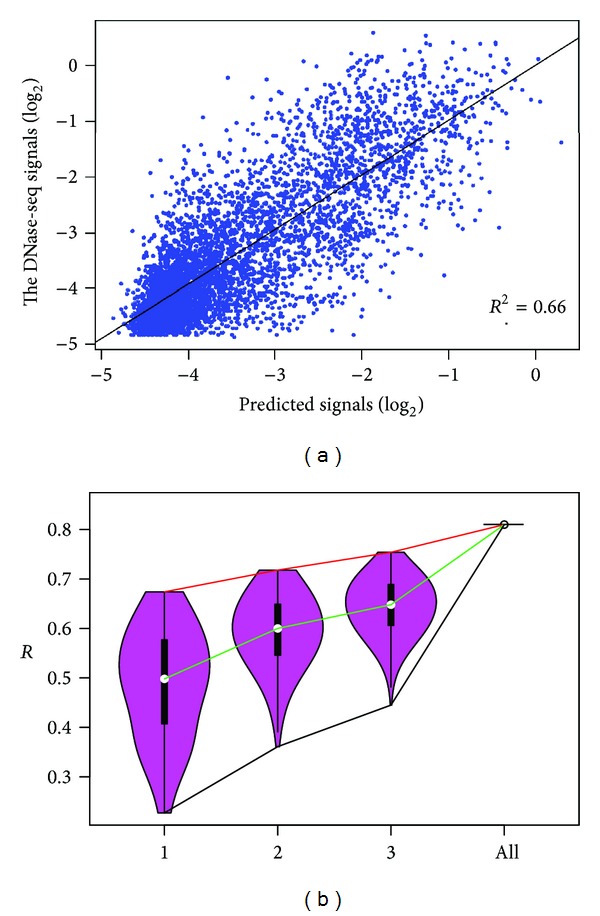
Redundancy of HM features and TF binding features. (a) Scatter plot of predicted versus experimentally measured DNase-seq signals using all HM and TF binding features. The black line represents the linear fit between predicted and measured signals (*R* ^2^, coefficient of determination). (b) Comparison of prediction powers (*R*, the square root of coefficient of determination) between all possible one-feature, two-feature, three-feature models, and the full model in H1hesc.

The previous analyses suggest that both histone modification features and transcription factor binding features are predictive for chromatin accessibility with high accuracy in DNase I hypersensitive sites. So, there is a question that whether the prediction power will increase if we use all these features. To address this question, we directly predicted the “openness” of chromatin using all features. As shown in Figure 4(a), the coefficient of determination (*R* ^2^ = 0.66) is a little higher (8%) than using only HM or TF binding features, which indicates that these two types of features are highly redundant. To check the importance of different features and their combinatorial effects, we tried to build models with all possible combinations of one to three features (Figure 4(b)). Focusing on the three-feature combinations (5456 models), we found that the least prediction power combinations (H3k36me3, H3k9me3, and ZNF274, *R* = 0.45) could achieve about 56% prediction power of the full model (*R* = 0.81). And there are 110 combinations achieving more than 90% prediction power of the full one. These analyses indicate that most of these features are highly redundant for chromatin accessibility. 

By examining the 110 high prediction power combinations, we found that seven chromatin features, H3k4me2, H3k4me3, H3k9ac, H4k20me1, SIN3A, ZNF143, SUZ12, were significantly enriched (*P* < 0.01, hypergeometric test) in the set of 110 models. Interestingly, all these features showed high prediction powers in the one-feature models except H4k20me1 and SUZ12. H4k20me1 is a particular one, which has been reported for the most predictive histone modification for gene expression [23]. SUZ12 is a part of Polycomb Repressive Complex 2 (PRC2) and may be involved in chromatin silencing with noncoding RNA [25]. The mechanisms of how SUZ12 influences chromatin structure are unknown; however, it may exert distinct impact on chromatin accessibility compared with other features.

### 3.5. Comparison with Other Models

In this study, we chose the SVR algorithm and the maximum signal in every hypersensitive region to model the relationship between chromatin features and chromatin accessibility. Generally, the SVR algorithm is a nonlinear regression method. We also have explored modeling using linear regression model and the average signal in every region. As shown in Table 1, prediction powers of models using average signal are significantly lower than the corresponding maximum signal models. And in either situation, the SVR models exhibit higher prediction power than linear models. Our results indicate that the “openness” of chromatin is determined by the maximum signal of features and their relationships are assumed as a nonlinear relevance.

## 4. Discussion 

In this work, we presented quantitative analyses of the relationship of histone modifications and the binding of transcription factors to chromatin accessibility separately and combinedly in DNase I hypersensitive sites. We first examined the percentage of feature peaks within DNase hypersensitive sites (DHSs) in human embryonic stem cell (H1hesc) line. We found that different chromatin features showed different location preference in DHS. This may be due to the particular function of different chromatin features. Thurman et al. have done similar analysis in K562 cell line [13] for TF binding features. In our analysis, we find that the percentage of transcription factors within DHS is significantly lower in the H1hesc cell line than that in K562 cell line. The reason may be as follows: in order to maintain the “stemness” state, most genes are repressed in the stem cell compared to the cancer cell line K562. This phenomenon means that the degree to which chromatin features occur in accessible chromatin may differ according to different cellular circumstances.

Our results demonstrate that both histone modification (HM) features and transcription factor (TF) binding features account for nearly 58% variance of chromatin accessibility in H1hesc cell line. For histone hallmarks, many activators of gene expression exhibited important impact on the “openness” of chromatin, such as H3k4me [21] and histone acetylations [28]. The hallmarks of repressors for gene expression such as H3k9me3 [21] show lower prediction powers. Unexpectedly, the transcription elongation hallmark H3k36me3 [29] shows the least prediction power. This is consistent with the viewpoint of a recently published paper. Chantalat et al. [22] argued that H3k36me3 is associated with constitutive and facultative heterochromatin. For TF binding features, the majority of TFs showed an important impact on chromatin accessibility except some transcriptional repressors, such as ZNF274 and SUZ12. This may indicate that the complex transcription process requires open chromatin environment [19].

It is generally accepted that cellular factors regulate the complex dynamic change of chromatin structure in a collective manner. We have shown that these features is highly redundant to predict chromatin accessibility and a small subgroup of features are able to achieve a very high prediction power. However, the mechanism of how these features cooperatively impact the openness of chromatin is still unclear, and we must note that our analysis could not reveal the “cause” or “consequence” relationship of HM and TF binding features to chromatin accessibility. Histone modifications play an important role in creating and maintaining the accessible chromatin environment [30] and may act as docking sites for transcription factors [31]. Some pioneer TFs tend to bind onto the genome and create an accessible site, such as FoxA1 [32] which is the best known pioneer transcription factor. Then, more transcription factors tend to bind onto the opening site and the DNase I hypersensitive site is created. As an extension, future work could explore the mechanisms of how these features cooperatively regulate open chromatin structure and their causal relationships, based on increased datasets.

## 5. Conclusion

We present genome-wide quantitative analysis of the impact of chromatin features to chromatin accessibility in DNase I hypersensitive sites. Our findings indicate that both histone modifications and the binding of transcription factors could explain nearly 58% variation of the “openness” of chromatin structure. The combinatorial effect analyses reveal that these chromatin features are highly redundant for prediction and H3k4me2, H3k4me3, H3k9ac, SIN3A, and ZNF143 show closest association with chromatin accessibility. Our results provide insights into the systematic effects of chromatin features to differential chromatin accessibility.

## Supplementary Material

Online Supporting Information S1. The prediction powers using all features with different sample sizes. In each sample size, we randomly sampled 500 times and modeling according to the procedure discussed in method 2.3 in each sample.
Online Supporting Information S2. The input maximum signal dataset including the maximum signals of Chip-seq and DNase-seq in 5000 DNase I hypersensitive sites.
Online Supporting Information S3. The input average signal dataset including the average signals of Chip-seq and DNase-seq in 5000 DNase I hypersensitive sites.
Online Supporting Information S4. The prediction powers (R) using only one feature.
Online Supporting Information S5. The prediction powers (R) using all possible two-feature combinations.
Online Supporting Information S6. The prediction powers (R) using all possible three-feature combinations.
Online Supporting Information S7. The related R codes are available.

## Figures and Tables

**Table 1:** Comparison of prediction powers with different models. The prediction power is represented as the square root of the coefficient of determination (*R*) for predicted and the actual DNase-seq signals. LM: linear regression model.

Model	SVR	LM	SVR	LM
	(max_signal)	(max_signal)	(avg_signal)	(avg_signal)
HM	0.76	0.69	0.70	0.56
TF	0.76	0.63	0.69	0.53
HM + TF	0.81	0.73	0.75	0.61

## Abbreviations

DHS: DNase I hypersensitive site
HM: Histone modification
SVR: Support vector regression.
