# Supplementary material for: A Quantitative Analysis of the Impact on Chromatin Accessibility by Histone Modifications and Binding of Transcription Factors in DNase I Hypersensitive Sites
Source: Biomed Res Int. 2013 Oct 22;2013:914971. doi: 10.1155/2013/914971 (PMC3819824; doi:10.1155/2013/914971)

Online Supporting Information S1. The prediction powers using all features with different sample sizes. In each sample size, we randomly sampled 500 times and modeling according to the procedure discussed in method 2.3 in each sample.

It can be seen that the prediction power increases with the rising of sample size. However, when the sample size reaches 2000, the prediction power increases only moderately. We chose 5000 as our sample size. The mean prediction power of 5000 sample size is 0.81±0.005 .

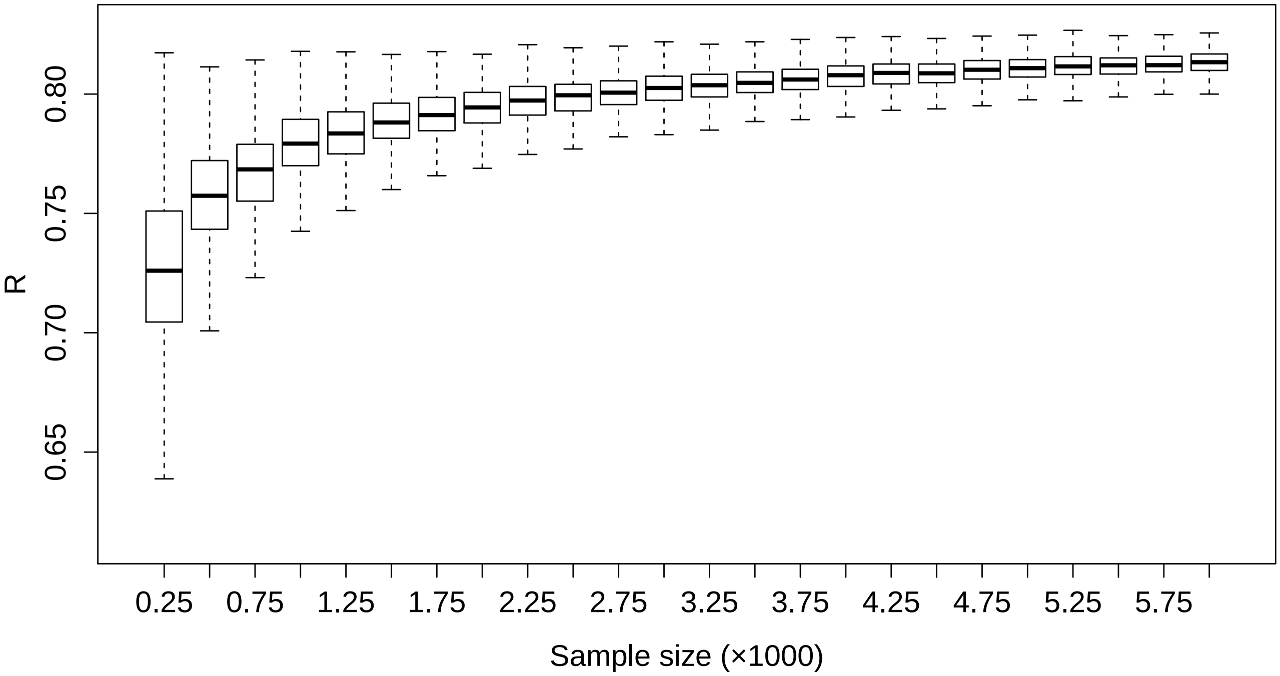

Supplement: Supplementary file 1 — Online Supporting Information S1. The prediction powers using all features with different sample sizes. In each sample size, we randomly sampled 500 times and modeling according to the procedure discussed in method 2.3 in each sample. Online Supporting Information S2. The input maximum signal dataset including the maximum signals of Chip-seq and DNase-seq in 5000 DNase I hypersensitive sites. Online Supporting Information S3. The input average signal dataset including the average signals of Chip-seq and DNase-seq in 5000 DNase I hypersensitive sites. Online Supporting Information S4. The prediction powers (R) using only one feature. Online Supporting Information S5. The prediction powers (R) using all possible two-feature combinations. Online Supporting Information S6. The prediction powers (R) using all possible three-feature combinations. Online Supporting Information S7. The related R codes are available. [file 914971.f1.zip › supple_S1.pdf]
